# Constipation and pain in Parkinson’s disease: a clinical analysis

**DOI:** 10.1007/s00702-023-02696-5

**Published:** 2023-10-28

**Authors:** Mohammad Al-Wardat, Piergiorgio Grillo, Tommaso Schirinzi, Chiara Pavese, Chiara Salimei, Antonio Pisani, Silvia Natoli

**Affiliations:** 1 Department of Rehabilitation Sciences, Faculty of Applied Medical Sciences, Jordan University of Science and Technology, Irbid, Jordan; 2 Department of Brain and Behavioral Sciences, University of Pavia, Pavia, Italy; 3 Unit of Neurology, Department of Systems Medicine, Tor Vergata University of Rome, 00133 Rome, Italy; 4 Department of Clinical-Surgical, Diagnostic and Pediatric Sciences, University of Pavia, Pavia, Italy; 5 Istituti Clinici Scientifici Maugeri IRCCS, Neurorehabilitation and Spinal Unit of Pavia Institute, Pavia, Italy; 6 Deptartment of Clinical Science and Translational Medicine, University of Rome Tor Vergata, Rome, Italy; 7 IRCCS Fondazione Mondino, Pavia, Italy

**Keywords:** Parkinson’s disease, Pain perception, Constipation, Non-motor symptom

## Abstract

Parkinson’s Disease (PD) is a neurodegenerative disorder characterized by both motor and non-motor symptoms (NMS). Among NMS, constipation and pain are both highly prevalent and debilitating affecting up to 80% of PD patients and impairing their quality of life. Here, we investigated the relationship between constipation and pain in PD patients. This is a retrospective study assessing the relationship between pain and constipation in a PD patient population from a clinical database of patients attending the outpatient clinic of the movement disorders division, Neurology Unit of Policlinico Tor Vergata, in Rome. Subjects were assessed with the Unified Parkinson’s Disease Rating Scale (UPDRS) part III, Hoehn and Yahr (H&Y) stage, King’s Parkinson’s Disease Pain Scale (KPPS), Brief Pain Inventory (BPI), Non-Motor Symptoms Scale (NMSS) and Beck Depression Inventory (BDI). Patients were further divided in two groups (Group 1, 32 patients with constipation and Group 2, 35 PD patients without constipation) ANOVA and ANCOVA analysis were used to compare the two groups. PD patients with constipation had significantly higher pain severity and pain interference, as measured by the BPI scale and higher total KPPS score, fluctuation-related pain, nocturnal pain, and radicular pain when compared to PD patients without constipation. This study highlights for the first time a possible interplay between constipation and pain in PD that deserves further investigations.

## Introduction

Parkinson’s Disease (PD) is a common neurodegenerative disorder whose clinical spectrum encompasses both motor signs and non-motor symptoms. The classical triad of bradykinesia, rigidity, and tremor is the cardinal element to suspect the diagnosis but represents only the tip of the iceberg. A big constellation of non-motor features, indeed, is also present in PD patients and silently impairs the quality of life (HRQoL) (Kalia and Lang 2015).

One of the most frequent and complaining non-motor symptoms is constipation, which affects up to 80% of PD patients, 20–30% in the early premotor phase (Picillo et al. 2021; Grillo et al. 2022). Although considered a condition simply associated with the disease, the latest evidence suggests that prodromal constipation might mark a specific PD neuropathological subtype (the so-called “body-first”) (Horsager et al. 2022; Grillo et al. 2022). Some authors speculate that in the “body-first” PD subtype, α-synuclein forms in the enteric nerve terminals or in the peripheral autonomic nervous system and then spreads via pons to the midbrain, thereby affecting non-dopaminergic nuclei along the route, including locus coeruleus and the raphe area, before substantia nigra is involved (Braak et al. 2003, 2004). As a fact, α -synuclein aggregates have been found in gastrointestinal nerve fibres years before Parkinson’s disease diagnosis (Stokholm et al. 2016). Noradrenaline produced in the brain underpins important aspects of sensorimotor processing and is involved in autonomic regulations. Hence, the early involvement of cerebral noradrenergic nuclei and the subsequent impairment of noradrenergic system may precede motor symptoms in PD and dominate PD-subtypes affected by pain and dysautonomia. The latter contributes to delayed gastric emptying and constipation (Ray Chaudhuri et al. 2023). Thus, despite prevalence of constipation in PD may vary according to the different diagnostic criteria used to define chronic constipation, the occurrence of this non-motor symptom prior to motor phase may reflect the early localization of Lewy pathology. Constipation is also a disturbance whose treatment with appropriate dietary regimens and laxatives can generally ameliorate patients’ quality of life (Stocchi and Torti 2017).

Also, pain is a common, highly disabling PD non-motor symptom (Joseph et al. 2019; Al-Wardat et al. 2022). The prevalence of pain ranges between 40 and 88%, with 5.5% of patients reporting it as the first symptom (Buhmann et al. 2020; Ghosh et al. 2020). The pathophysiological mechanisms of pain in PD are complex and multicausal, and the available medications (e.g., non-steroidal anti-inflammatory drugs (NSAIDs), opioids, etc.) often produce only a temporary relief (Buhmann et al. 2020; Ghosh et al. 2020) and their use is limited by toxicity and adverse effects.

Chronic pain may be related or unrelated to PD. Among PD-related pain types, musculoskeletal conditions, typically affecting these patients, have been implicated as pain generators (Mylius et al. 2021). However, altered pain processing appears to be relevant in the development of chronic pain in PD. Indeed, abnormalities of neurotransmitters involved in PD pathophysiology may contribute to impair endogenous pain modulation (Mylius et al. 2009).

Constipation has been positively associated with pain severity in chronic pain patients (Shiro et al. 2017; Arai et al. 2018; Frazzitta et al. 2019). Authors suggest that constipation may relate to chronic pain for several reasons, such as age, gender, BMI, insufficient physical activity and excessive sedentary behaviour. Moreover, the use of opioid analgesics can contribute to the slow the gut transit by binding mu-opioid receptors in the enteric nervous system and thereby inhibiting motor and secretomotor neurons of the gut. Intriguingly, constipation is associated to gut microbiome dysbiosis and some authors speculate that gut dysbiosis can alter neuropeptide production in the brain and subsequently impair endogenous pain modulation (Arai et al. 2018). Of interest, studies on subjects with chronic abdominal pain and healthy volunteers recently demonstrated that constipation and pain may influence each other, suggesting that treating one may help to alleviate the other and vice versa (Shiro et al. 2017; Chen et al. 2021). Albeit this might represent an intriguing strategy even for PD patients, the association between slow intestinal transit and altered nociception in PD has not been assessed yet.

Here we investigated the phenomenology of pain in two well-characterized cohorts of PD patients, one with constipation and one without, in order to discuss the potential relationship between gastrointestinal motility and pain perception.

## Methods

### Study design

This is a retrospective study assessing the relationship between pain and constipation in a PD patient population. The participants were attending the outpatient clinic of the movement disorders division, Neurology Unit of Policlinico Tor Vergata, Rome, Italy. The study has been conducted in accordance with the ethical principles of Helsinki and approved by the local EC (protocol number 0026092/2017). All participants signed the informed consent.

### Eligibility criteria and data collection

We included in the study patients with PD evaluated at the outpatient clinic of the movement disorders division of the University Hospital Policlinico di Tor Vergata, Roma between 2021 and 2022. Inclusion criteria were: PD diagnosis according to 2015 MDS criteria (Postuma et al. 2015), stable drug treatment while in the ON medication phase. Exclusion criteria were: severe systematic diseases such as pulmonary disorders, heart failure, chronic liver, renal failure, infectious disease, gastrointestinal disorders, and chronic inflammatory disease were excluded.

All patients underwent a neurological evaluation conducted by a neurologist specialized in movement disorders. The clinical data and evaluations were obtained via a standardized personal interview. All enrolled patients were receiving the best medical treatment available. We recorded the following clinical and demographic variables: age, disease duration, MDS – Unified PD Rating Scale (UPDRS) part III (Postuma et al. 2015), Hoehn and Yhar stage (HY) (Hoehn and Yahr 2001), Kings Parkinson’s Pain Scale (KPPS, total and sub-items scores) (Chaudhuri et al. 2015), Brief pain inventory (BPI) (Caraceni et al. 1996), Non-motor symptoms scale (NMSS) (Chaudhuri and Martinez-Martin 2008), Hamilton depression scale (HAM-D) (Pavan 1982), and Mini Mental State Examination (MMSE) (Folstein et al. 1975) adjusted for age and educational level, levodopa equivalent daily dose (LEDD) (Tomlinson et al. 2010). The presence of constipation was defined according to the Rome IV criterion of constipation (Lacy et al. 2016).

### Statistical analysis

The distribution of variables was evaluated with the Shapiro–Wilk test. Non-normally distributed continuous variables were (Log10) + 1-trans- formed. Group differences were assessed by analysis of variance (ANOVA). To compare the KPPS, BPI, and domain scores between the two groups, an analysis of covariance (ANCOVA) was conducted, adjusted for potential confounding factors (H&Y staging, disease duration, MDS-UPDRS-III and LEED). Significance was set at *p* < 0.05. Statistical analysis was performed on the blind by using IBM- SPSS-26. Data are available from authors upon reasonable request.

## Results

We included in the study 67 PD patients. According to the Rome IV criterion of constipation, the participants were distributed in two groups: Group 1 included 32 PD patients with constipation and Group 2 included 35 PD patients without constipation. The demographic and clinical features of the two groups are presented in Table 1. All patients were on stable treatment with a dopaminergic medication and exhibited good motor compensation in their limb movements. None of the patients displayed any psychiatric or cognitive impairments. MMSE scores ranged normal in both groups. There were no significant differences in age, gender, disease duration, and MMSE between the two groups. However, patients with constipation had a significantly longer disease duration and higher scores in MDS-UPDRS III, H&Y stage, and LEDD. Patients from both groups reported one or more types of pain based on the KPPS score, being musculoskeletal, fluctuation, nocturnal, and radicular pain the most frequent.

**Table 1:** Demographical and clinical characteristics of the patients

Variable	PD with constipation (*n* = 32)	PD without constipation (*n* = 35)	*P* value
Sex (m/f)	12/20	11/24	n.s
Age (y)	63.97 (7.25)	60.43 (8.42)	n.s
PD onset (y)	6.50 (5.25)	3.97 (2.39)	n.s
LEDD (mg/day)	678.91 (304.48)	406.94 (215.02)	** < 0.001**
MDS-UPDRS-part III	30.19 (11.61)	20.23 (8.03)	** < 0.001**
HY	2.41 (0.57)	1.96 (0.57)	** < 0.001**
MMSE	27.36 (1.74)	28.23 (1.80)	n.s
HAM-D (depression)	18.19 (7.28)	12.97 (6.97)	**0.01**

Statistical significance is marked in bold

*PD*: Parkinson’s disease, *m*: male, *f:* female, *y*: years, *LEDD*: levodopa equivalent daily dose, *UPDRS* Unified PD Rating Scale, *HY* Hoehn and Yhar stage, *MMSE* Mini mental status exam, *HAM-D* Hamilton depression scale

**Fig. 1:**
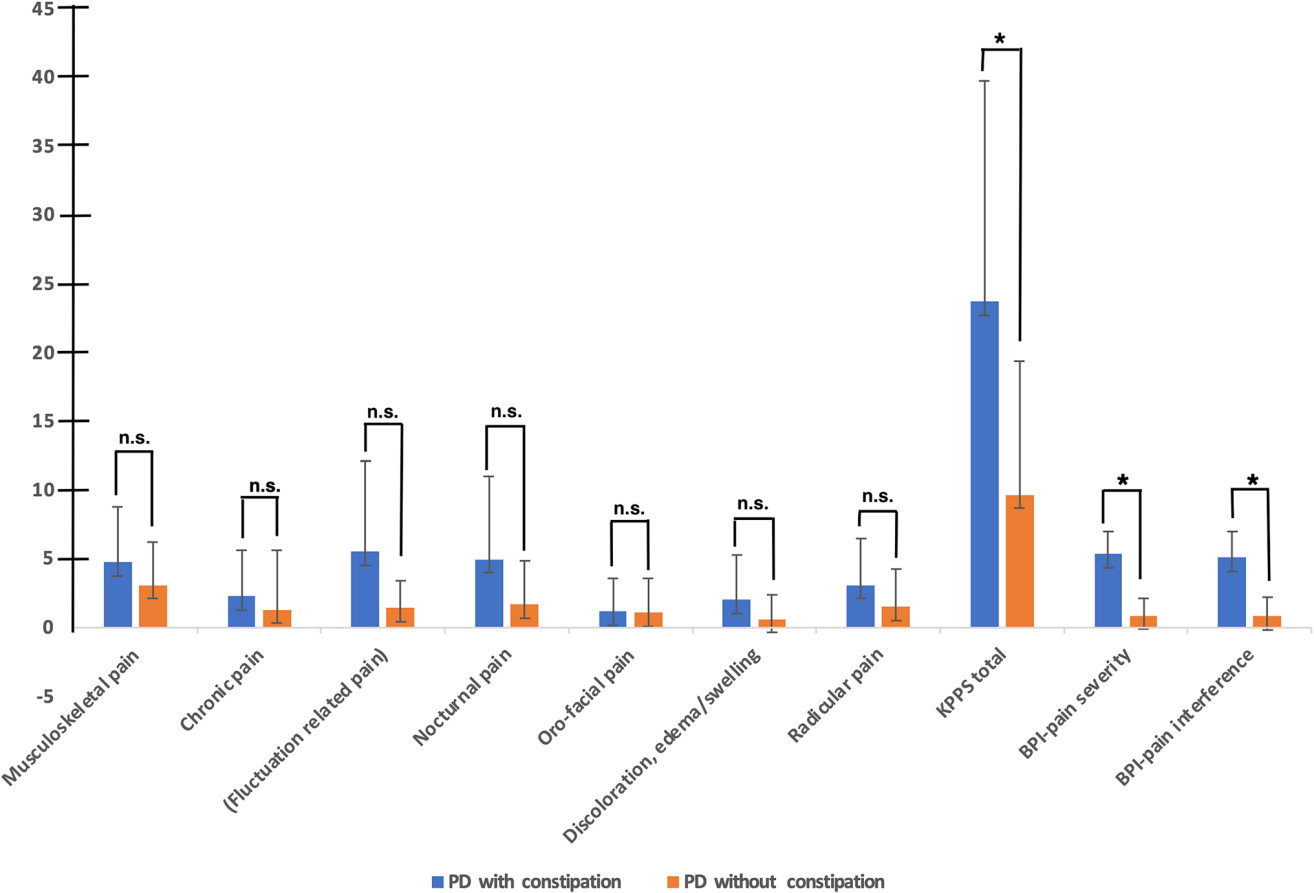
The differences and average of Brief Pain Inventory (BPI) scores and King Parkinson’s pain scale (KPPS) total scores and sub-domains, represent as (Mean ± SD) in PD patients with constipation and without. ANCOVA test adjusted for H&Y staging, LEED, MDS-UPDRS-III and disease duration. The significance values are reported (*P < 0.05)

Pain differences between PD with and without constipation on BPI and KPPS total and subscale are shown in Fig. 1. PD patients with constipation had significantly higher pain severity and interference, as measured by the BPI scale compared to PD patients without constipation, while there was no significant difference in the severity of pain types in the two groups. In addition, the means of KPPS total, fluctuation-related pain, nocturnal pain, and radicular pain were significantly higher in the PD with constipation compared to PD without. The ANOVA results showed significant differences in pain intensity and interference between the PD with constipation and without F(1, 64) = 152.24, *p* = 0.001, F(1, 64) = 107.12, *p* = *0.001,* respectively. Furthermore*,* both groups showed a significant differences in KPPS total scores F(1, 65) = 19.09, *p* = *0.001,* chronic pain F(1, 65) = 12.42, *p* = *0.001, Fluctuation related pain* F(1, 65) = 7.45, *p* = *0.008,* discoloration, edema/swelling F(1,65) = 4.9*, p* = *0.03,* and radicular pain F(1, 65) = 4,38*, p* = *0.04*. ANCOVA test showed a significant difference in KPPS total scores independently from H&Y staging, LEED, MDS-UPDRS-III, and disease duration (*P* = 0.001). Additionally, ANCOVA test showed a significant difference in the BPI pain severity and interferences independently from H&Y staging, LEED, MDS-UPDRS-III, and disease duration, respectively (*P* = 0.001, *P* = 0.001).

**Fig. 2:**
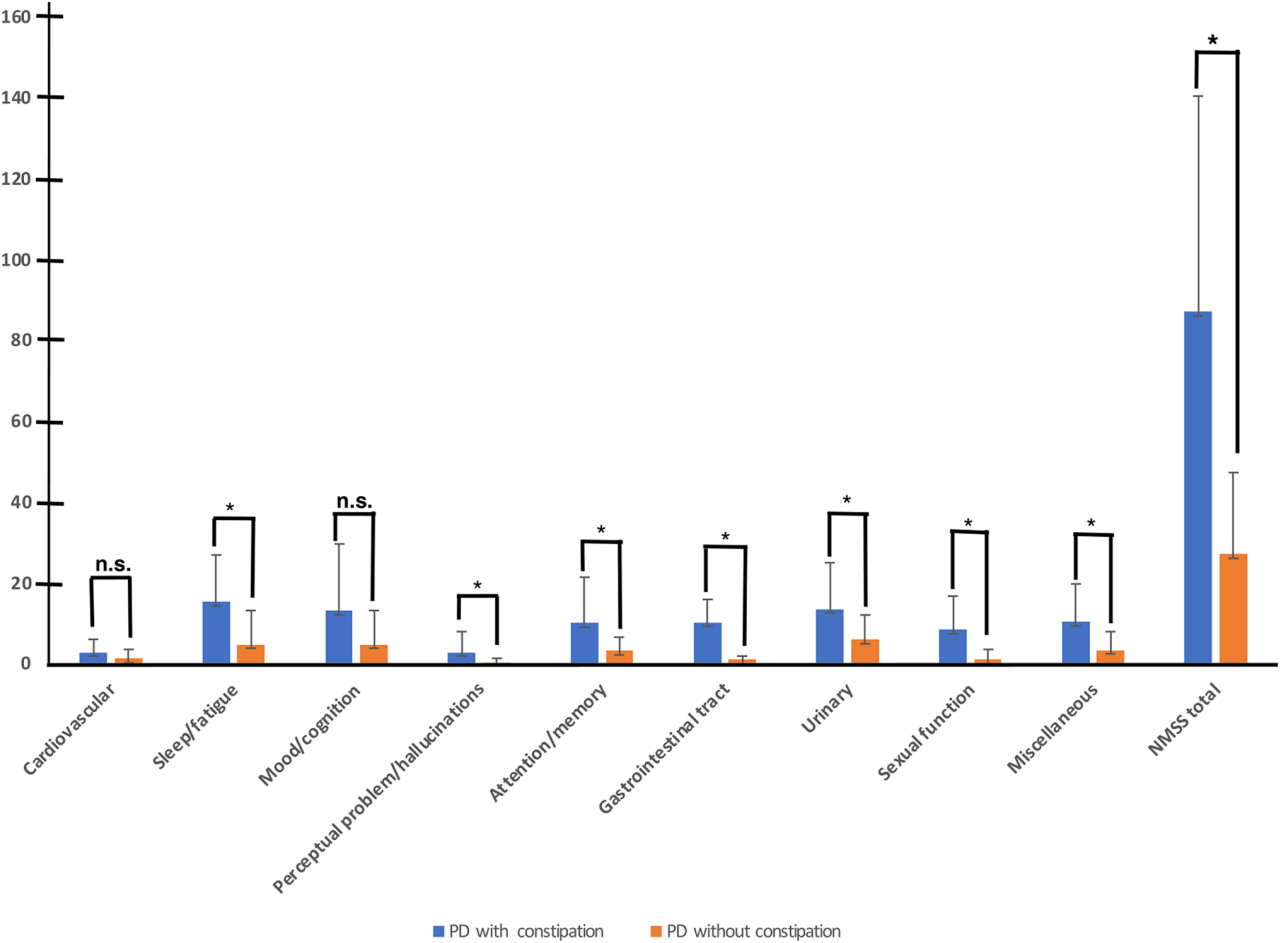
The differences and average of Non-motor Symptoms Scale (NMSS) scores and sub-domains, represent as (Mean ± SD) in PD patients with constipation and without. The significance values are reported (*P < 0.05)

We further explored the relation between NMSS and constipation by comparing the two groups. The ANOVA analysis revealed significant differences in total NMSS scores (*P* = 0.001) and sub domains (sleep and fatigue, perceptual problem/ hallucination, attention/memory/gastrointestinal tract, urinary, sexual function, and miscellaneous *p* < 0.001) except cardiovascular domain (*P* = 0.06) and mood/cognition domain (*P* = 0.07) between PD with constipation and without (Fig. 2). These findings support the idea that constipation may be a marker of more severe non-motor symptoms in PD. Moreover, in our cohort of patients ANCOVA test showed a non-significant difference between groups with regard to the NMSS total scores or sub-domains, except for constipation (*P* = 0.001), when data are adjusted for H&Y staging, LEED, MDS-UPDRS-III, and disease duration.

## Discussion

In this study, we evaluated pain perception through BPI and KPPS in two different cohorts of PD subjects, respectively with and without constipation, with the aim of exploring a possible connection between these frequent and disabling non-motor disturbances. We found that PD patients with constipation compared to those without- scored significantly higher in BPI-severity, BPI-interference and KPPS total. Our analysis suggests that constipation is significantly associated with pain in PD.

Interestingly, previous studies revealed a significant association between constipation and pain severity among patients with chronic pain (Shiro et al. 2017; Arai et al. 2018; Frazzitta et al. 2019). These studies investigated potential mechanistic links contributing to the association between constipation and pain severity, highlighting the significance of physical activity level, and endogenous pain modulatory mechanisms as key factors. Constipation correlates with inadequate levels of physical activity and an excess of sedentary behavior (Alves Silva et al. 2020). Conversely, reduced physical activity, has been associated with impaired gastrointestinal motility and increased risk of constipation (Peters et al. 2001). Likewise, physical inactivity could lead to increased pain sensitivity due to changes in musculoskeletal health (Law and Sluka 2017). It is extensively recognized that individuals with PD exhibit lower levels of physical activity in comparison to healthy individuals (von Rosen et al. 2021). Thus, despite constipation in PD basically reflects the degeneration of the enteric nervous system by the Lewy Body pathology (Grillo et al. 2022), physical inactivity may contribute to it. Constipation may long precede the onset of motor symptoms, suggesting that, at least in some cases, neurodegeneration may proceed bottom-up, from the enteric or peripheral autonomic nervous system to the brain (the so called “body-first” hypothesis) (Horsager et al. 2022). Recent works showed that the “body first” PD subtype with prodromal constipation displays more severe clinical burden in motor or cognitive domains (Picillo et al. 2021; Grillo et al. 2022). Here, we show that constipation may also be associated with other non-motor symptoms, i.e. pain, and therefore it should be considered a clinical marker of greater frailty in PD.

Our analysis shows that patients with constipation had a significantly longer disease duration. This is in line with the progressive nature of PD. However, the multivariate analysis, shows a significant difference in KPPS total scores and BPI independently from disease duration. Thus, the association of pain with constipation may not reflect a “more severe” PD due to progression of neurodegeneration. Instead, it may reflect an interplay between the two conditions (pain and constipation), or overlapping, and still poorly understood mechanisms, that account for the correlation that we have found.

Nociception is a complex process that involves multiple neural structures including nociceptors, dorsal horn of spinal cord, spinothalamic system, and cortical regions. Neurodegenerative changes in PD can potentially alter this process at both central and peripheral level, thus generating different subtypes of pain that often coexist in the same subject (Antonini et al. 2018; Rukavina et al. 2019). Indeed, pain related to PD may be primary (i.e. nociplastic, that is a maladaptive pain processing within the central nervous system), or secondary to musculoskeletal or visceral conditions, or neuropathic in origin (Mylius et al. 2021). Pain is exacerbated by dopamine fluctuations in PD patients, supporting the idea that dopamine depletion has an underlying role in pain hypersensitivity in PD, although the exact mechanism is still unclear. Moreover, there is increasing evidence that a hypodopaminergic tone characterizes chronic pain states (Taylor et al. 2016). PD patients in the off-phase display lower pain thresholds and tolerance compared to healthy controls, underpinning increased spinal nociceptive activity, and decreased descending inhibition (Gerdelat-Mas et al. 2007; Mylius et al. 2009). Pain thresholds are partially restored, but not completely normalised, during on-states after dopamine medication, suggesting that additional mechanisms are likely to contribute to pain hypersensitivity in PD (Thompson et al. 2017). Recently, it has been suggested that noradrenaline dysfunction may also contribute to alter the pain endogenous modulation in PD (Ray Chaudhuri et al. 2023) Indeed, in healthy individuals, pain descending pathways inhibit spinal nociceptive activity through the actions of noradrenaline at α2-adrenoceptors, whereas spinal noradrenergic activity is impaired in chronic pain states (Bannister and Dickenson 2017). It is also likely that noradrenergic transmission is negatively involved in dysautonomic symptoms in PD (Titova et al. 2017).

From a pathophysiological point of view, there may be other overlaps in the in the genesis of pain, constipation, and PD. Indeed, the three conditions have been individually associated with gut microbial dysbiosis (Wallen et al. 2020; Pan et al. 2022).

Gut microbiome alterations can drive maladaptive neuroplasticity in pain processing pathways by inducing immune cells to release pro-inflammatory factors and ultimately amplify dorsal root ganglion and dorsal horn neuronal excitability (Liu et al. 2023), Moreover, gut microbiome is necessary for absorption and production of the amino acid tryptophan, which is the precursor of serotonin (5-HT), a neurotransmitter involved in pain modulation (Bannister and Dickenson 2017), as well as in the regulation of mood and cognition (Cryan and Leonard 2000), which are often impaired in chronic pain states and in PD. Widespread chronic pain correlates to reduced 5-HT levels, and reduced tryptophan absorption (Lattanzio 2017). Likewise, the dysfunction of the serotonergic system is involved in many aspects of PD (Hsam and Kohl 2023). Furthermore, 5-HT is involved in the local regulation of gut movements (Mawe and Hoffman 2013) and its depletion may contribute to the alteration of gut motility in PD.

Thus, the degeneration of dopaminergic and non-dopaminergic pathways in the brain, characteristic of PD, may impact the intricate balance of neurotransmitters involved in pain modulation and gut functionality (Maiti et al. 2017) and impair central nociceptive processing and modulation, playing an important role in pain pathogenesis in PD (Fil et al. 2013; Buhmann et al. 2017; Antonini et al. 2018; Blanchet and Brefel-Courbon 2018). Dopamine fluctuations worsen pain perception in PD (Mylius et al. 2009). Our findings indicate that PD patients with constipation are more prone to fluctuation-related and nocturnal pain which may be consequent to an insufficient bioavailability of dopaminergic drugs.

It is well known that an altered gastrointestinal motility and changes in the microbiota composition can alter levodopa pharmacokinetics (Zhong et al. 2023). A stable gut microbiota, indeed, contributes to the regulation of multiple neuro-chemical and neuro-metabolic pathways that guarantee a normal gut physiology and proper transmission along the gut-brain axis. By contrast, ever-increasing evidence demonstrates that imbalance within gut microbiome may be involved in PD pathogenesis, gastrointestinal disorders, and pain processing alterations (Bravo et al. 2012; Shiro et al. 2017; Freidin et al. 2021; Chen et al. 2021; Warnecke et al. 2022). In the complex interplay of constipation, impaired levodopa absorption and likely intestinal dysbiosis and impaired pain processing, the relationship between constipation and increased pain occurrence is not surprising. Constipation-related fluctuations in levodopa absorption may worsen motor complications in PD patients and levodopa-fluctuations related dyskinesia and dystonia may also result in painful sensations (Stocchi and Torti 2017).

Despite PD was initially described as a motor syndrome associated to dopamine deficiency, it is now well recognized that NMS have a greater impact on Health-related quality of life (HRQOL) than motor symptoms (Barone et al. 2009). However, furthers subanalysis of single NMS domains suggest that neither pain nor constipation primarily contributes to HRQoL, despite these finding are not uniform. Inconclusive results may depend on lack of specific and shared definitions and scoring systems for pain and constipation in PD. If not carefully investigated, pain in PD may be neglected during routine visits. In fact, patients often do not report spontaneously on pain (Buhmann et al. 2020). The awareness that PD patients with constipation are at higher risk of experiencing pain, should lead clinicians to perform a more careful assessment of pain in this group of patients; furthermore, it should prompt efforts to restore gastrointestinal motility and levodopa absorption as an important step to manage pain in this population, with hope-for reducing painkiller and NSAID overuse. In addition, patients should be educated to modify their fiber and water intake and to change sedentary habits in order to manage constipation (Astarloa et al. 1992; Alwardat et al. 2019). We should recognize that the relationship between pain and constipation likely involves a complex interplay of multiple factors, also including phycological aspects. Thus, actions aiming at restoring well-being in a patient-centered proactive strategy, by promoting physical activity, dietary habits, and by managing anxiety and depression, may all potentially affect HRQoL, reducing psychosocial distress (Schirinzi et al. 2019, 2020; Pontone et al. 2023). These are all recognised factors that deserve further exploration in future studies.

There are several limitations to be acknowledged in this study. First, the sample size is limited to the number of patients who fulfilled the inclusion criteria from a previous database that was created for different purposes. Therefore, these data should be confirmed in an *ad-hoc* powered study. Second, the retrospective nature of the study and the absence of long-term follow-up could affect the ability to determine the temporal relationship between constipation and pain severity in PD. A longitudinal study design would be more appropriate to assess the long-term relationship between these variables. Third, we did not consider that levodopa is a possible cause of constipation and related gastrointestinal disorders, therefore, patients on OFF-medication state should be included in future studies.

## Conclusion

Overall, our results suggest that constipation is associated with increased pain severity and interference in individuals with PD, as well as specific types of pain such as fluctuation-related pain and nocturnal pain. Despite the limitations, this study highlights for the first time the importance of considering constipation as a potential contributing factor to pain in PD patients. However, future studies may investigate the possible interplay between constipation, pain and HRQol in PD patients.

## Data Availability

The datasets generated during and/or analysed during the current study are not publicly available due to participants’ privacy and ethical concerns but are available from the corresponding author at a reasonable request.
